# The porcine circovirus 3 humoral response: characterization of maternally derived antibodies and dynamic following experimental infection

**DOI:** 10.1128/spectrum.00870-24

**Published:** 2024-06-25

**Authors:** Molly Kroeger, Gun Temeeyasen, Steven Dilberger-Lawson, Eric Nelson, Ronaldo Magtoto, Luis Gimenez-Lirola, Pablo Piñeyro

**Affiliations:** 1 Department of Veterinary Diagnostic and Production Animal Medicine, Iowa State University, Ames, Iowa, USA; 2 Department of Veterinary and Biomedical Sciences, South Dakota State University, Brookings, South Dakota, USA; University of Georgia, Athens, Georgia, USA

**Keywords:** Porcine Circovirus 3, humoral response, maternal immunity

## Abstract

Since Porcine Circovirus 3 (PCV3) was first identified in 2016, our understanding of the humoral response is still relatively scarce. Current knowledge of the PCV3 humoral response is primarily based on field studies identifying the seroprevalence of PCV3 Cap-induced antibodies. Studies on the humoral response following experimental PCV3 infection have conflicting results where one study reports the development of the Cap IgG response 7 days postinfection with no concurrent Cap IgM response, while a second study shows a Cap IgM response at the same time point with no detection of Cap IgG. The dynamics of the PCV3 Cap and Rep IgG following maternal antibody transfer and experimental infection have not been well characterized. Additionally, the cross-reactivity of convalescent serum from PCV2 and PCV3 experimentally infected animals to serologic methods of the alternate PCV has limited evaluation. Here, we show that maternally derived antibodies were detectable in piglet serum 7–9 weeks postfarrowing for the Cap IgG and 5-weeks-post farrowing for the Rep IgG using Cap- and Rep-specific enzyme linked immunosorbent assays (ELISA) and immunofluorescent assays (IFA) methods. Following experimental inoculation, Cap IgG was detected at 2-weeks-post inoculation and Rep IgG detection was delayed until 4-weeks-post inoculation. Furthermore, convalescent serum from either PCV2 or PCV3 methods displayed no cross-reactivity by serological methods against the other PCV. The information gained in this study highlights the development of both the Cap- and Rep-specific antibodies following experimental infection and through the transfer of maternal antibodies. The increased understanding of the dynamics of maternal antibody transfer and development of the humoral response following infection gained in the present study may aid in the establishment of husbandry practices and potential application of prophylactics to control PCV3 clinical disease.

**IMPORTANCE:**

Research on Porcine Circovirus 3 (PCV3) immunology is vital for understanding and controlling this virus. Previous studies primarily relied on field observations, but they have shown conflicting results about the immunological response against PCV3. This study helps fill those gaps by looking at how antibodies develop in pigs, especially those maternal-derived, and their impact in neonatal pigs preventing PCV3-associated disease in piglets. In addition, we look at the dynamics of antibodies in experimental infections mimicking infection in pigs in the grower-phase condition. Understanding this process can help to develop better strategies to prevent PCV3 infection. Also, this research found that PCV2 and PCV3 do not cross-react, which is crucial for serological test development and results interpretation. Overall, this work is essential for improving swine health and farming practices in the face of PCV3 infections.

## INTRODUCTION

Circoviruses are small, circular, single-stranded DNA viruses in the family *Circoviridae* and in the genus *Circovirus* (1). Currently, four species of porcine circoviruses (PCV) have been identified that naturally infect pigs. PCV1 was identified in 1974 as a cell culture contaminant in the porcine kidney (PK-15) cell line (1) and is nonpathogenic to pigs (2, 3). In contrast, PCV2 was identified in the 1990s and is the primary etiological agent of porcine circovirus-associated disease (PCVAD), and since that time, PCV2 has become one of the most economically impactful and important swine diseases globally (4–6). PCV3 was identified in 2016 and has most commonly been associated with multisystemic inflammation, reproductive failure, and subclinical infection (7, 8). Most recently, PCV4 was identified in 2019 in pigs with respiratory and enteric clinical signs (9); however, the role of PCV4 as a causative agent remains controversial due to the presence of coinfections and limited studies using direct detection methods (7, 10).

The PCV3 genome comprises 2,000 base pairs (bp) with two major open reading frames (ORFs) oriented in opposite directions. ORF1 encodes the 297 amino acid (aa) Replicase (Rep) protein, which is involved in viral replication. Additionally, ORF2 encodes for the 214 aa Capsid (Cap) protein, which is the sole component of the viral capsid and gives the virus many of its antigenic properties (8, 11, 12). Similarly, the PCV2 genome is 1,766–1,768 bp (13) with a 315 aa Rep and 233–234 aa Cap protein (14, 15). PCV2 and PCV3 have relatively low nucleotide homology at the whole genome (46.8%) and ORF2 (26%–36%) levels (8, 12, 16). The PCV4 genome is 1,770 bp with a 296 aa Rep and 228 aa Cap protein (9). PCV4 has relatively low nucleotide homology to the PCV2 whole genome (51.5%) and ORF2 (12.7%–45.0%) as well as the PCV3 whole genome (42.9%–45.0%) and ORF2 (23.2%–24.8%) (9, 17).

Several enzyme-linked immunosorbent assays (ELISA) and indirect immunofluorescent assays (IFA) have been developed to detect PCV3 Cap-specific antibodies. The antigens platforms reported for the development of PCV3 ELISAs include the full length, codon optimized Cap protein expressed in a baculovirus (18) and *Escherichia coli* expression systems (19). The 33 N-terminus aa of the Cap protein composes a nuclear localization signal (NLS) with a high arginine content and rare codons, resulting in decreased protein expression in prokaryotic expression systems (20). Thus, most ELISAs utilize a truncated Cap protein, which excludes the NLS sequence expressed in *E. coli* (21–23). IFA assays have been developed by expressing the Cap in baculovirus (24) and other eukaryotic expression systems (25).

Current knowledge of the PCV3 humoral response is primarily based on field studies identifying the seroprevalence of PCV3 Cap-induced antibodies (21). Retrospective seroprevalence studies have reported the PCV3 antibody positivity rate ranging from 20% to 80% (18, 21). Notably, serological evaluations conducted in sow farms did not find significant differences within farm seroprevalence rate or antibody titer measured by baculovirus-expressed capsid protein-based indirect ELISA regardless of the presence of clinical signs. Additionally, the antibody positivity rate was significantly higher in sows with high levels of viremia compared to sows with low level of viremia (18). Transfer of maternal antibodies has been hypothesized based on the absence of viremia in animals of 3–4 weeks of age (26) and increased detection of viremia in animals 6–24 weeks of age (27). However, the dynamics of PCV3-maternal antibodies have not been characterized. Studies on the humoral response following experimental PCV3 infection have reported conflicting results. One study reported the development of the Cap IgG response 7 days postinfection with no concurrent Cap IgM response (28), while a second study showed a Cap IgM response at the same time point with no detection of Cap IgG (22). Moreover, experimental studies have not characterized the Rep antibody dynamic or the protective role conferred by the humoral response. Given the genetic similarity of PCV2 and PCV3 (8, 12, 16), the potential of antibody cross-reactivity has been speculated following natural infection and vaccination. PCV3 convalescent serum has shown no cross-reactivity to PCV2 virus-like particles (21); however, the cross-reactivity of PCV2 and PCV3 serum to serological methods of the alternate PCV has not been reported.

The humoral response to following the transfer of maternal antibodies and development of antibodies after natural infection has been well characterized resulting in the development of specific vaccination schedules to greatly reduce PCVAD. Both PCV2 Cap and Rep maternally derived antibodies have been detected in piglets with the highest titers observed at 1 week of age (29), and the mean half-life of PCV2 Cap maternally derived antibodies is 19 days. In 3-week old specific pathogen free pigs experimentally inoculated with PCV2, Cap IgG, and Rep antibodies were detectable 7–28 days postinfection (dpi) with neutralizing titers detectable 10 dpi (30). Furthermore, experimental inoculated cesarean-derived colostrum-deprived (CD/CD) 8-week-old pigs produced Cap antibodies earlier and at a higher titer compared to Rep antibodies (31).

The understanding of the PCV3 humoral response remains limited. Thus, an increased understanding of the dynamics of maternal antibody transfer and development of the humoral response following infection may aid in the establishment of husbandry practices and potential application of prophylactics to control PCV3 clinical disease. Therefore, the objectives were to develop PCV3 Cap and Rep IFA and ELISA methods to characterize the PCV3 IgG antibody dynamics following the transfer of maternal antibodies and experimental infection. Furthermore, the cross-reactivity of convalescent serum from PCV2 and PCV3 experimentally infected animals to serologic methods of the alternate PCV was evaluated. The information gained in this study highlights the development of both the Cap- and Rep-specific antibodies following experimental infection and through the transfer of maternal antibodies. Additionally, convalescent serum from either PCV2 or PCV3 methods displayed no cross-reactivity by serological methods against the other PCV.

## MATERIALS AND METHODS

### Study 1: Maternal transfer of antibodies from naturally infected sows

A cross-sectional evaluation of pregnant sows 2 weeks before farrowing was performed in a gestational unit located in Iowa, USA. Assuming default 50% seroprevalence with 95% confidence interval and 17% absolute error, the sample size was calculated to be 30 sows (*n* = 30) as previously described (32, 33). The farm had a PCV3 history characterized by an abortion storm with a 40% abortion rate for approximately 1 month. The presence of PCV2, porcine reproductive and respiratory syndrome virus (PRRSV), and porcine parvovirus 1 (PPV1) was ruled out by PCR, and presence of PCV3 was confirmed by PCR conducted on fetal tissue by routine diagnostic submission at the ISU-VDL. Sow sera were evaluated by a PCV3 ORF2 recombinant protein-based indirect ELISA (22). Three litters consisting of a total of 31 piglets (litter 1, *n* = 8; litter 2, *n* = 11; and litter 3, *n* = 12) were arbitrarily selected for the longitudinal study from litters of sow with low, mid, and high pre-farrow antibodies levels relative to the distribution of ELISA *S*/*P* ratios of 30 sows selected for pre-farrow prescreening. Each piglet was individually identified with plastic ear-tags. All piglets remained with the dams for 21 days (±3 days), and no cross-fostering was performed during lactation to ensure specific antibody transfer from dams. At weaning, all piglets were reallocated into a nursery barn and commingled with piglets from the same farm and an additional sow farm. Blood samples were collected once a week from 1 to 9 weeks of post farrowing using a single‐use blood collection system (Vacutainer SST, BD, Franklin Lakes, NJ, USA). Blood samples were kept on ice during transport to the ISU-VDL. The serum was separated immediately upon arrival to the laboratory by centrifugation at 2,000 *g* for 15 min, aliquoted, and stored at −80°C until final serological and molecular testing.

### Study 2: PCV3 antibody dynamic following experimental inoculation

The inoculation study was carried out as previously described (28). Briefly, 18 5-week-old CD/CD pigs were randomly assigned to four treatment groups: PCV3 infectious material (PCV3, *n* = 6), PCV3 infectious material+adjuvant (keyhole limpet hemocyanin) (PCV3+KLH, *n* = 6), adjuvant control (KLH control, *n* = 3), and mock-inoculated negative control (negative control, *n* = 3). The PCV3 and PCV3+KLH groups were inoculated bilaterally and intramuscularly in the neck with 2 mL of tissue homogenate with Ct = 8 (gc = 3.38 × 10^12^ mL^−1^), and inoculated intranasally with 2 mL of tissue homogenate with Ct = 13 (gc = 1.04 × 10^11^ mL^−1^), and re-inoculated 7 days later following the same protocol. Both control groups were inoculated similarly to the challenged groups with 2 mL of Minimum Essential Medium Eagle (MEM) at 0 dpi, and reinoculated at 7 dpi. Immunostimulation was performed in the PCV3+KLH and KLH Ctrl groups by subcutaneous administration of 2 mL of keyhole limpet hemocyanin (1 mg mL^−1^) (KLH, Sigma-Aldrich, St. Louis, MO, USA) emulsified in incomplete Freund’s adjuvant (ICFA, Sigma-Aldrich) into the right hip and right shoulder (1 mL/site) at 3 dpi and reinoculated in the left hip and left shoulder at 7 dpi with the same KLH dose. One pig from the PCV3 and PCV3+KLH groups was randomly euthanized at 11 dpi. Furthermore, one animal per treatment group was euthanized at 21 dpi, and all remaining animals were euthanized at 42 dpi. Blood was collected from all pigs at 0, 3, 7, 10, 14, 17, 21, 24, 28, 31, 35, and 42 dpi in 5 mL serum separator tubes (Thermo Fisher Scientific, Waltham, MA, USA). The blood was centrifuged at 2,000 *g* for 10 min, and serum aliquots were stored at −80°C until testing.

### Study 3: PCV2 infection study

Ten 10-day-old CD/CD pigs were sham inoculated with an adjuvant carrier for a PCV2 vaccine efficacy trial 49 days pre-challenge (pre chal). All pigs were challenged at 59 days of age with PCV2 tissue homogenate (2 mL intranasal and 1 mL intramuscular) on 0 days post challenge (dpc). Blood was collected from all pigs on 49 days pre chal and 0, 14, and 28 dpc in 5 mL serum separator tubes (Thermo Fisher Scientific, Waltham, MA, USAThermo Fisher Scientific). The blood was centrifuged at 2,000 *g* for 10 min, and serum aliquots were stored at −80°C until testing.

### PCV3 qPCR

In study 1, viremia was evaluated by litter where serum samples were pooled with a maximum of five animals per pool. In study 2, viremia was evaluated individually. PCV3 detection was performed by quantitative-PCR (qPCR) as previously described (34) according to standard operating procedure at the Iowa State University Veterinary Diagnostic Laboratory (ISU-VDL). Briefly, MagMAX-96 Pathogen RNA/DNA kit (Applied Biosystem, Waltham, MA, USA) with a KingFisher Flex 96 Deep-Well Magnetic Particle Processor (Thermo Fisher Scientific) was used to extract the DNA from pooled serum according to the manufacturer’s instructions. A conserved region of the PCV3 replicase (ORF1) gene was detected from the extracted DNAs by qPCR using the TaqMan Fast Virus 1-Step Master Mix (Life Technologies, Carlsbad, CA, USA) with forward primer 5′-TGTWCGGGCACACAGCCATA-3′ and reverse primer 5′-TTTCCGCATAAGGGTCGTCTT-3′ with the probe 5′-/5SUN/ACCACAAAC/ZEN/ACTTGGCTC/31ABkFQ/−3′ previously described (34). qPCR was performed on 7500 Fast Real-Time PCR System (Applied Biosystems, Foster City, CA, USA) with the following cycling conditions: one cycle at 50°C for 5 min, one cycle at 95°C for 20 s, 40 cycles at 95°C for 3 s, and 60°C for 30 s. All samples were controlled appropriately with positive, negative, and internal controls according to validated ISU-VDL standard operating procedures. Samples with Ct < 37.0 were considered positive.

### Production of PCV3 recombinant Cap and Rep proteins

Recombinant truncated Cap and full-length Rep proteins were used as antigens for the indirect ELISAs based on the ORF1 and ORF2 sequences of the PCV3 29160 (GenBank KT869077). A truncated Cap gene (bp 90–645) was used as previous studies have shown exclusion of the nuclear localization sequence improves prokaryotic expression (20). The truncated Cap and full-length Rep sequence were codon optimized for prokaryotic expression and synthesized with flanking *BamHI* and *XhoI* endonuclease restriction sites in the pET-28b expression vector, which includes a 6× histidine tag on the N terminus (GenScript, Piscataway, NJ, USA). The synthesized plasmids were transformed into BL21(DE3) *E. coli* cells (New England Biolabs, Ipswich, MA, USA). Bacterial clones were grown in Luria-Bertani (LB) broth (Thermo Fisher Scientific, Waltham, MA, USA) containing 50 µg/mL kanamycin at 37°C with shaking at 250 rpm. At an A_600_ of 0.5, protein expression was induced using 1.0 mM isopropyl β-D-1 thiogalactopyranoside (IPTG, Thermo Fisher Scientific, Waltham, MA, USA) and incubated for an additional 8 h at 37°C. Bacterial cultures were harvested by centrifugation at 12,000 *g* for 10 min at 4°C and were resuspended in solution (B-PER, Thermo Fisher Scientific, Waltham, MA, USA). The suspension was centrifuged at 12,000 *g* and the insoluble Rep protein was denatured using 8 M urea. Both Cap and Rep proteins were purified using nickel-nitrilotriacetic acid (NTA) affinity column chromatography (Qiagen, Hilden, GER) (35). The purified proteins were analyzed for purity and linear integrity by 12% sodium dodecyl sulfate-polyacrylamide gel electrophoresis (SDS-PAGE). To confirm specificity, the proteins were recognized by Western blotting using an anti-histidine monoclonal antibody (Thermo Fisher Scientific, Waltham, MA, USA).

### Indirect PCV3 Cap ELISA

An Immulon 2HB, 96-well plate (Thermo Fisher Scientific, Waltham, MA, USA) was coated with 100 µL the truncated Cap protein diluted 1:1,000 in antigen coating buffer (15 mM sodium carbonate 35 mM sodium bicarbonate, pH 9.6) and incubated for 1 h at 37°C and overnight at 4°C. The next morning, plates were washed three times with PBST (phosphate-buffered saline containing 0.1% Tween 20) and were blocked with 200 µL per well of blocking buffer (PBS containing 1% Casein and 0.05% Tween 20) for 1 h at 37°C. Plates were washed three times with PBST and 100 µL of swine serum samples tested in duplicated were diluted 1:50 in blocking buffer was added and incubated for 1 h at room temperature. Next, plates were washed three times with PBST and 100 µL per well goat anti-pig IgG horseradish peroxidase (HRP) secondary antibody (Bethyl Laboratories, Inc., Montgomery, TX, USA) diluted 1:2,500 in blocking buffer was added and incubated for 1 h at room temperature. Plates were washed three times with PBST, and 100 µL per well of tetramethylbenzidine-hydrogen peroxide (TMB) substrate-solution (Invitrogen, Waltham, MA, USA) was added and incubated for 7 min at room temperature. 100 µL per well ELISA stop solution (Thermo Fisher Scientific, Waltham, MA, USA) was added, and reactions were measured for optical density (OD) at 450 nm using as ELISA plate reader (BioTek Instruments, Inc., Winooski, VT, USA) operated with commercial software (Gen5, BioTek Instruments, Inc.).

Each plate contained a positive control (Pig 8, 28 dpi, Study 1) and negative control (Pig 1, 0 dpi, Study 1) tested in duplicate. Serum antibody responses were expressed as a sample-to-positive (*S*/*P*) ratios: *S*/*P* ratio = (sample OD – negative control mean OD)/(positive-control mean OD – negative control mean OD). For study 2, the estimated positive cutoff *S*/*P* ratio was calculated as the mean *S*/*P* ratios of the negative controls’ plus three standard deviations for each time point and then taking the mean of the individual time points (28, 36).

### Indirect PCV3 Rep ELISA

An Immulon 1B, 96-well plate (Thermo Fisher Scientific, Waltham, MA, USA) was coated with 100 µL the Rep protein diluted 1:1,000 in antigen coating buffer (15 mM sodium carbonate 35 mM sodium bicarbonate, pH 9.6) and incubated for 1 h at 37°C and overnight at 4°C. The next morning, plates were washed three times with PBST and were blocked with 200 µL per well of blocking buffer (PBS containing 1% Casein and 0.05% Tween 20) for 1 h at 37°C. Plates were washed three times with PBST and 100 µL of swine serum samples tested in duplicated were diluted 1:50 in blocking buffer was added and incubated for 1 h at room temperature. Next, plates were washed three times with PBST and 100 µL per well goat anti-pig IgG HRP secondary antibody (Bethyl Laboratories, Inc.) diluted 1:10,000 in blocking buffer was added and incubated for 1 h at room temperature. Plates were washed three times with PBST and 100 µL per well of TMB substrate-solution (Invitrogen) was added and incubated for 7 min at room temperature. 100 µL per well ELISA stop solution (Thermo Fisher Scientific, Waltham, MA, USA) was added and reaction OD was measured at 450 nm using as ELISA plate reader (BioTek Instruments, Inc.) operated with commercial software (Gen5, BioTek Instruments, Inc.).

Each plate contained a positive control (Pig 17 at 35 dpi, Study 1) and negative control (Pig 3, 0 dpi, Study 1) performed in duplicate. Serum antibody responses were expressed as *S*/*P* ratios as described in previous section. For study 2, the estimated positive cutoff *S*/*P* ratio was calculated as the mean *S*/*P* ratios of the negative controls’ plus three standard deviations for each time point and then taking the mean of the individual time points (28, 36).

### PCV3 Cap and Rep IFAs

The full-length Cap and Rep sequences from PCV3 29160 (KT869077) were cloned into a pcDNA3.1(+)-N-6His expression vector using the *BamHI* and *XhoI* endonuclease restriction sites (GenScript). The plasmids were transformed into and purified from DH5α *E. coli* cells (New England Biolabs).

The Cap-pcDNA3.1 and Rep-pcDNC3.1 expression vectors were transfected into Human Embryonic Kidney (HEK) 293 cells (ATCC, Manassas, VA, USA) using Lipofectamine 3000 (Invitrogen) according to manufacturers recommended instructions. After 48 h incubation, cells were fixed with 80% acetone. Transfection and expression of the Cap and Rep proteins were confirmed by staining with an anti-histidine monoclonal antibody diluted 1:1,000 in PBS (Thermo Fisher Scientific) followed by a Fluorescein isothiocyanate (FITC) conjugated goat anti-mouse secondary antibody diluted 1:100 in PBS (Thermo Fisher Scientific, Waltham, MA, USA). For the Cap IFA, swine serum samples were tested in duplicate and were diluted two-fold starting at 1:40 (Study 1) or 1:4 (Study 2) in blocker (PBS containing 1% bovine serum albumin) at 50 µL per well and were incubated for 1 h at 37°C. For the Rep IFA, swine serum samples were tested in duplicate and were diluted two-fold starting at 1:10 (Study 1) or 1:4 (Study 2) in blocker at 50 µL per well and were incubated for 1 h at 37°C. Plates were washed three times with PBS and 50 µL per well of FITC conjugated anti-swine IgG secondary (Bethyl Laboratories, Inc.) diluted 1:100 in blocker was added and incubated for 1 h at 37°C. Plates were washed three times with PBS and staining was observed by fluorescent microscopy (Olympus IX83 Research Inverted Microscope, Olympus Life Science, Waltham, MA, USA). The IFA titer was expressed as the highest dilution where fluorescent signal was detected.

### PCV2 qPCR, ELISA, and IFA

PCV2 qPCR, ELISA, and IFA were conducted according to standard operating procedure at the ISU-VDL. For the PCV2 qPCR, MagMAX-96 Pathogen RNA/DNA kit (Applied Biosystem, Waltham, MA, USA) with a KingFisher Flex 96 Deep-Well Magnetic Particle Processor (Thermo Fisher Scientific, Waltham, MA, USA) was used to extract the DNA from pooled serum according to the manufacturer’s instructions. DNA extracts were used to detect the conserved region of the PCV2 replicase (ORF1) gene using TaqMan Fast Virus 1-step Master Mix (Life Technologies, MA, USA) with forward primer 5′-GACTGTWGAGACTAAAGGTGGAACTGTA-3′ and reverse primer 5′-GCTTCTACACCTGGGACAGCA-3′ with the probe 5′-/56-FAM/-CCCGTTGGAATGGT/3MGBEc/−3′. The qPCR was performed (7500 Fast Real-Time PCR System, Applied Biosystems, Foster City, CA, USA) with the following cycling conditions: one cycle at 50°C for 5 min, one cycle at 95°C for 20 s, 40 cycles at 95°C for 3 s and 60°C for 30 s. Samples with Ct values < 37 were considered positive.

The Ingezim PCV2 IgG ELISA kit (Gold Standard Diagnostics, Budapest, Hungary) was utilized for PCV2 ELISA testing. Briefly, 100 µL per well of diluted serum samples were added to the ELISA plate and incubated for 1 h at room temperature. Plates were washed four times with wash solution, and 100 µL per well of conjugate was added and incubated for 30 min at room temperature. Plates were washed six times with wash solution, and 100 µL per well of substrate was added. Plates were incubated for 10 min at room temperature, and 100 µL of stop solution was added. Sample OD was measured at 450 nm within 5 min after the addition of the stop solution. Positive and negative cutoffs and sample *S*/*P* ratios were calculated according to manufacture formulas.

For the PCV2 IFA, PK-15 cells were mixed with PCV2 and seeded in a 96-well plate. Following 72 h incubation, plates were fixed with 80% acetone and stored at −70°C. Serum samples were diluted two-fold from 1:20 to 1:2,560 in PBS and 90 µL of each serum dilution per well was added to warmed plate. Plates were incubated for 1 h at 37°C, and plates were washed three times with PBS. 50 µL per well of conjugate was added and incubated for 30–60 min at 37°C. The IFA titer was expressed as the highest dilution where fluorescent signal was detected compared to the positive control.

### Statistical analysis

Differences in IFA titers and ELISA *S*/*P* ratios for studies 1 and 2 were analyzed by 2-way ANOVA with Tukey’s correction for multiple comparisons. Statistical significance was set with an alpha value of 0.05. Data analysis was performed using GraphPad Prism (GraphPad Software, La Jolla, CA, USA).

## RESULTS

### Expression of PCV3 Cap and Rep recombinant proteins

**FIG 1:**
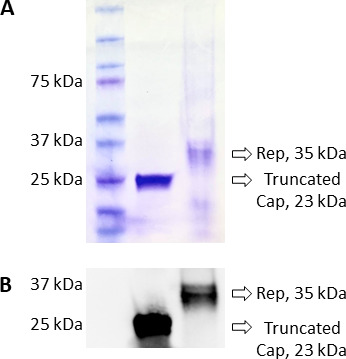
Purification and specificity of the truncated Cap and full-length Rep proteins of PCV3. (A) Coomassie blue staining of *E. coli* expressed and Nickel-NTA affinity column chromatography purified truncated Cap (left lane, 23 kDa) and full-length Rep protein (right lane, 35 kDa). (B) Western blotting confirming specificity and predicted molecular masses of the truncated Cap (left lane) and full-length Rep (right lane) polyhistidine tagged proteins by staining with an anti-histidine monoclonal antibody.

The truncated Cap and full-length Rep of PCV3 were cloned and expressed in *E. coli* as a polyhistidine fusion protein. Protein purity was analyzed using SDS-PAGE, where both the truncated Cap and Rep proteins migrated to the predicted molecular masses of 23 kDa (truncated Cap) and 35 kDa (Rep) by Coomassie brilliant blue 250 staining (Fig. 1A). Further analysis by Western blot using anti-histidine monoclonal antibody showed specificity and proteins with the predicted molecular masses (Fig. 1B).

### *In vitro* expression of PCV3 Cap and Rep proteins

**FIG 2:**
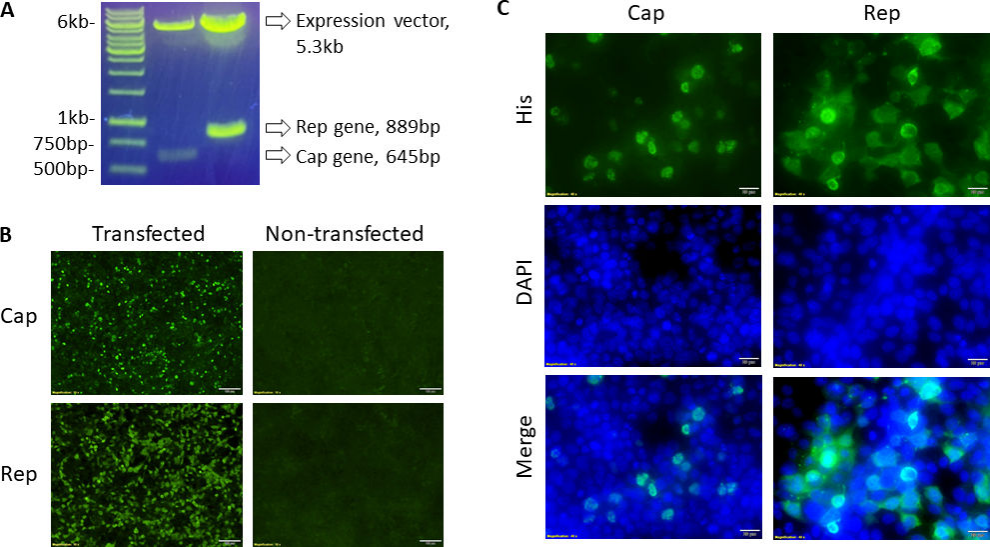
Molecular cloning and *in vitro* expression of the full-length Cap and Rep proteins of PCV3. (A) 1% agarose gel of Cap-pcDNA3.1(+)-N-6His (left lane) and Rep-pcDNA3.1(+)-N-6His (right lanes) plasmids digested with *BamHI* and *XhoI* endonuclease restriction enzymes. Band sizes appear as predicted: Cap (645 bp), Rep (889 bp), and expression vector (5.3 kb). (B) *In vitro* expression of Cap and Rep proteins in transfected HEK 293T cells by staining with an anti-histidine monoclonal primary antibody followed by an FITC conjugated goat anti-mouse secondary observed under ultraviolet fluorescence at 10× magnification. (C) Characterization of Cap and Rep proteins in transfected HEK 293T cells by staining with an anti-histidine monoclonal primary antibody followed by an FITC conjugated goat anti-mouse secondary antibody and DAPI observed under ultraviolet fluorescence and DAPI filter at 40× magnification.

The full-length Cap and Rep genes of PCV3 were cloned into the pcDNA3.1(+)-N-6His expression vector. Enzyme restriction analysis by *BamHI* and *XhoI* endonuclease of the Cap-pcDNA3.1(+)-N-6His and Rep-pcDNA3.1(+)-N-6His plasmids on 1% Agarose gel confirmed successful cloning of the Cap (645 bp) and Rep (889 bp) genes into the expression vector (5.3 kb) (Fig. 2A). *In vitro* expression of the Cap and Rep proteins in transfected HEK 293T cells was confirmed by detection of green-fluorescent intranuclear and intracytoplasmic staining, respectively (Fig. 2B). The signal/background discrimination from transfected cell versus cell-control was evaluated for both the Cap and Rep proteins, and the transfection rate was approximately 70% (Fig. 2B). Dual staining with anti-histidine and DAPI (4′,6-diamidino-2-phenylindole) in transfected cells revealed expression of the Cap protein in nucleus while the Rep protein was predominately expressed in the cytoplasm (Fig. 2C).

### Characterization of PCV3 Cap and Rep maternal antibody transfer

**FIG 3:**
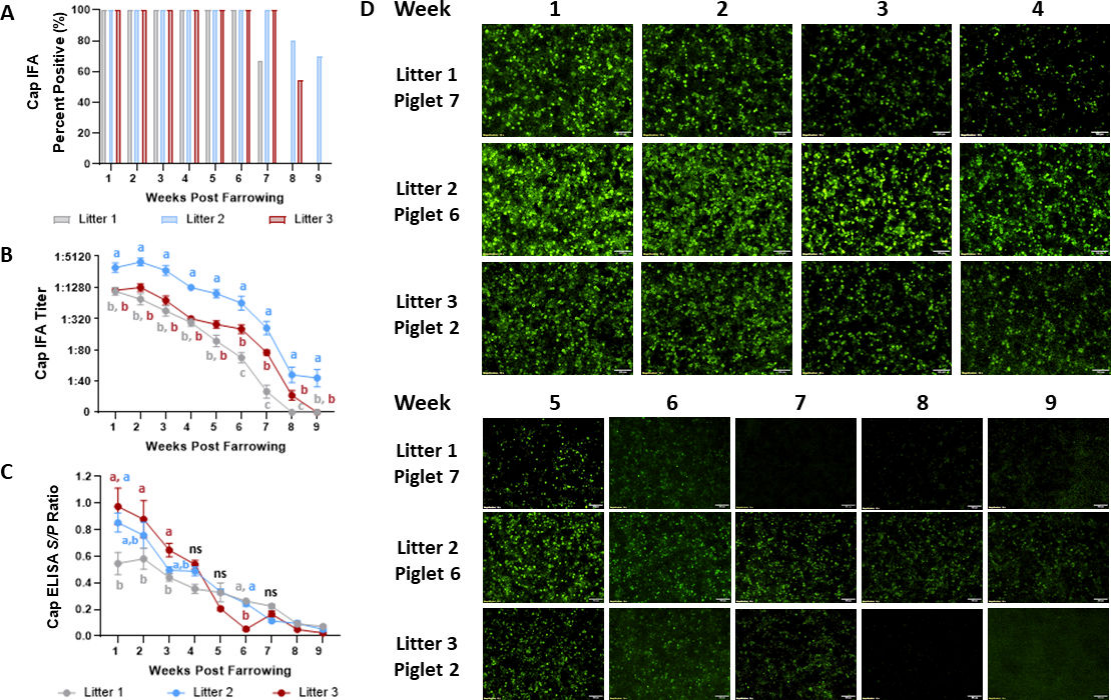
Kinetic of the maternally transferred serum Cap IgG antibodies by IFA and ELISA in piglets 1–9 weeks post farrowing. Three litters of piglets born from commercial sows naturally exposed to PCV3 were selected for study. The Cap IgG antibody status was evaluated by the percentage of piglets per litter positive (A), average IFA titer by litter (B), and average ELISA *S*/*P* ratios (C). IFA and ELISA methods concurred maximal Cap IgG titers occurred 1–2 weeks post farrowing, which waned in the following weeks. Antibodies were detectable 9-weeks post farrowing by IFA in litter 2 piglets but were nondetectable in litter 1 and 3 piglets. (D) The Cap IgG IFA staining from one representative piglet per litter at a serum dilution of 1:20 and 4× magnification, where the bright and diffuse immunofluorescent staining decreased over time correlating to lower IFA titers. Bars represent the SE of the mean, and significance was established at *P* < 0.05.

Thirty sows from a commercial sow farm with a history of PCV3 had a positive Cap antibody status 2 weeks prior to before farrowing by ORF2 recombinant protein-based indirect ELISA (data not shown) (22). The humoral response was evaluated longitudinally in three litters of piglets (31 total piglets) 1–9 weeks post farrowing using Cap and Rep indirect ELISA and IFA. All piglets from all litters showed detectable Cap IgG antibodies by IFA through 6 weeks post farrowing. Furthermore, Cap IgG antibodies were detectable in approximately 60% of litter 2 piglets by 9 weeks post farrowing while no Cap IgG antibodies were detected in piglets of litters 1 and 3 at the same time point (Fig. 3A and B). The highest Cap IgG IFA antibody titers and ELISA *S*/*P* ratios occurred 1–2 weeks post-farrowing in all piglets and declined to nearly undetectable levels between 7 and 9 weeks of age (Fig. 3B and C). Litter 2 had significantly higher IFA titers (*P* < 0.05) compared to litters 1 and 3 from 1 to 9 weeks post farrowing with litter 2 IFA titers reaching 1:5,120 at 1-week post farrowing. No significant differences in IFA titer for litters 1 and 3 were observed between 1 and 5 weeks post farrowing with maximum titers of 1:1,280 at 1-week post farrowing. The Cap IgG IFA staining of one representative piglet per litter is shown in Fig. 3D, where positive antibody detection is characterized by nuclear green-fluorescent staining of transfected cells.

**FIG 4:**
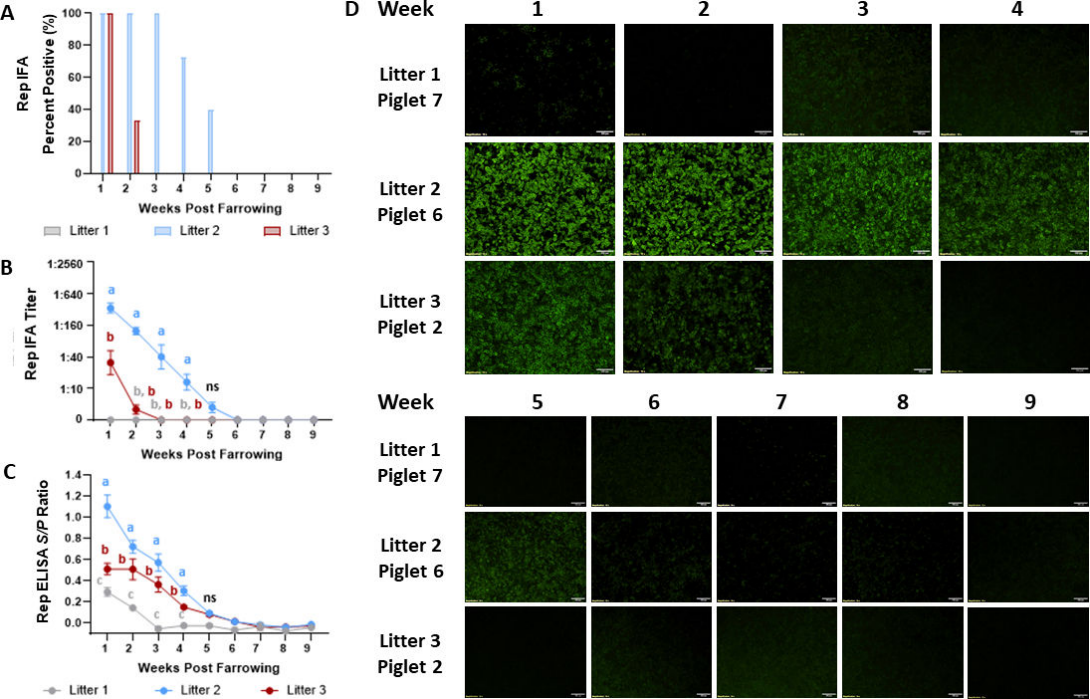
Kinetics of the maternally transferred serum Rep IgG antibodies by IFA and ELISA at 1–9 weeks post farrowing. Three litters of piglets born from commercial sows naturally exposed to PCV3 were selected for the study. The Rep IgG antibody status was evaluated by the percentage of positive piglets per litter by IFA (A), average IFA titer by litter (B), and average ELISA *S*/*P* ratios (C). Rep IgG antibodies were nondetectable in litter 1 piglets. For litter 2 and 3 piglets, Rep IgG antibodies were at maximal levels by IFA and ELISA 1-week post farrowing and rapidly declined to nondetectable levels by 3-weeks post farrowing for litter 3 piglets and 6-weeks for litter 2 piglets. (D) The Rep IgG IFA staining from one representative piglet per litter at a serum dilution of 1:10 and 4× magnification, where no staining is evident in piglet 7 from litter 1 and the bright and diffuse immunofluorescent staining is not visible past 6 weeks post farrowing for piglet 6 from litter 2. Bars represent the SE of the mean, and significance was established at *P* < 0.05.

For the Rep IgG response, no antibodies were detected in litter 1 piglets by IFA. However, Rep IgG antibodies were detectable by IFA in 100% of piglets in litters 2 and 3 1-week post farrowing (Fig. 4A). Rep IgG antibody levels declined more rapidly compared to Cap IgG antibodies, with no detectable Rep IgG antibodies by 3-weeks post farrowing for litter 3 and 6-weeks post farrowing for litter 2 (Fig. 4A through C). The highest Rep IgG IFA antibody titers and ELISA *S*/*P* ratios were observed at 1-week post farrowing. IFA titers and ELISA *S*/*P* ratios in litter three more rapidly declined to a negative antibody status 2-weeks post farrowing. In contrast, the IFA titer and ELISA *S*/*P* ratios remained significantly higher until 5-weeks post farrowing (Fig. 4B and C). The highest Rep IgG IFA titer was 1:640 for piglets in litter 2 1-week post farrowing, which was approximately eight-fold lower than the maximum Cap IgG titer observed at the same time point. The Rep IgG IFA staining of one representative piglet per litter is shown in Fig. 4D. No immunofluorescent staining was observed in piglet 7 from litter 1. Furthermore, the cytoplasmic green-fluorescent staining of transfected cells in the piglets from litters 2 and 3 rapidly decreases and is not visible after 6 weeks post farrowing (Fig. 4D).

The PCV3 viremia, as detected by qPCR, was evaluated by litter from 1 to 9 weeks post farrowing. No detectable viremia was observed in litter 1 throughout the duration of the study. From 1 to 4 weeks post farrowing, litters 2 and 3 had intermittent detection of PCV3 DNA at relatively low amounts with a Ct values ranging from 32.3 to 36.9. Interesting, litter 3 had a low amount of PCV3 (Ct = 36.7) detected in the serum 9-weeks post farrowing after six consecutive weeks of having no detectable viremia (Table 1).

**TABLE 1:** Detection of PCV3 DNA in the serum of piglets born from naturally infected sows 1–9 weeks post farrowing^*a*^

Litter	Weeks of age (serum) (Ct-value)								
	1	2	3	4	5	6	7	8	9
1	−	−	−	−	−	−	−	−	−
2	+ (36.8)	+(36.4)	−	+ (36.9)	−	−	−	−	−
3	+ (35.5)	−	+ (32.3)	−	−	−	−	−	+ (36.7)

^*a*^ Serum samples were pooled by litter with a maximum of five animals per pool. The mean of the pooled Ct values by litter were calculated to yield the litter Ct value.

### Characterization of PCV3 Cap and Rep antibody dynamics in grower pigs following experimental infection

**FIG 5:**
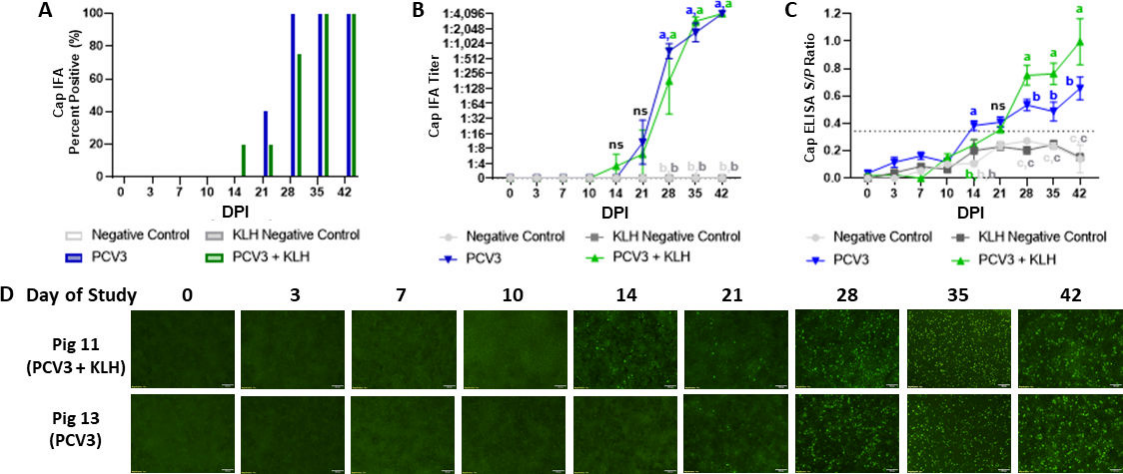
Dynamics of Cap IgG antibodies by IFA and ELISA in CD/CD pigs experimentally inoculated with PCV3 tissue homogenate with and without immunostimulation. PCV3 and PCV3+KLH groups were inoculated intranasally with 2 mL of PCV3 tissue homogenate (1.04 × 10^11^ genome copies mL^−1^) and intramuscularly with 2 mL of PCV3 tissue homogenate (3.38 × 10^12^ genome copies mL^−1^) then reinoculated 7 days after the first inoculation. Immunostimulation was performed in the PCV3+KLH and KLH negative control groups through subcutaneous administration of 1 mL of 1 mg mL^−1^ of KLH in ICFA. Cap IgG antibodies were evaluated by the percentage of pigs per group (A), average IFA titer by group (B), and average ELISA *S*/*P* ratios by group (C). Seroconversion was detected at 14 dpi with significantly antibody higher levels in the PCV3 and PCV3+KLH groups by IFA and ELISA occurring at 28–42 dpi. (D) The Cap IgG IFA staining from one representative pig in the PCV3 and PCV3+KLH groups at a serum dilution of 1:4 and 4× magnification, where relatively low amounts of immunofluorescent staining are present at 14 dpi for pig 11 in the PCV3+KLH group and much greater amounts of bright and diffuse immunofluorescent staining from both pigs are present 28–42 dpi, correlating to the increase in IFA titers. Bars represent the SE of the mean, and significance was established at *P* < 0.05.

The evaluation of the antibody dynamic in experimentally infected pigs showed a Cap IgG antibody response, detectable in a single pig as early as 14 dpi in the PCV3+KLH group and two pigs at 21 dpi in the PCV3 group. All the pigs from both PCV3-inoculated groups were positive by IFA at 35 dpi (Fig. 5B). All pigs in both PCV3 and PCV3+KLH-inoculated groups had significantly higher Cap IgG antibody titers by IFA, compared with negative control groups, by 28 dpi and reached a maximum IFA titer of 1:4,096 by 42 dpi (Fig. 5A and B). The IFA antibody titers and ELISA *S*/*P* ratios for the PCV3 and PCV3+KLH group were significantly higher (*P* < 0.05) than the negative control and KLH negative control groups 28–42 dpi (Fig. 5B and C). No statistical difference between the PCV3 and PCV3+KLH group IFA titers was observed. However, the statistical analysis of the ELISA *S*/*P* ratios showed that the PCV3+KLH group had significantly higher levels of antibodies (*P* < 0.05) compared to the PCV3 group at 28–42 dpi (Fig. 5C). All the pigs in the mock-inoculated negative control and adjuvant control groups tested negative for the presence of Cap IgG by IFA and ELISA throughout the study duration (Fig. 5B and C). The Cap IgG IFA staining of one representative pig from the PCV3 and PCV3+KLH groups is shown in Fig. 5D. Relatively low amounts of nuclear green-fluorescent staining were present at 14 dpi for pig 11 in the PCV3+KLH group and 21 dpi for pig 13 in the PCV3 group. The nuclear green-fluorescent staining was more intense in both pigs between 28 and 42 dpi, correlating to the increase in IFA titers and ELISA *S*/*P* ratios (Fig. 5D).

**FIG 6:**
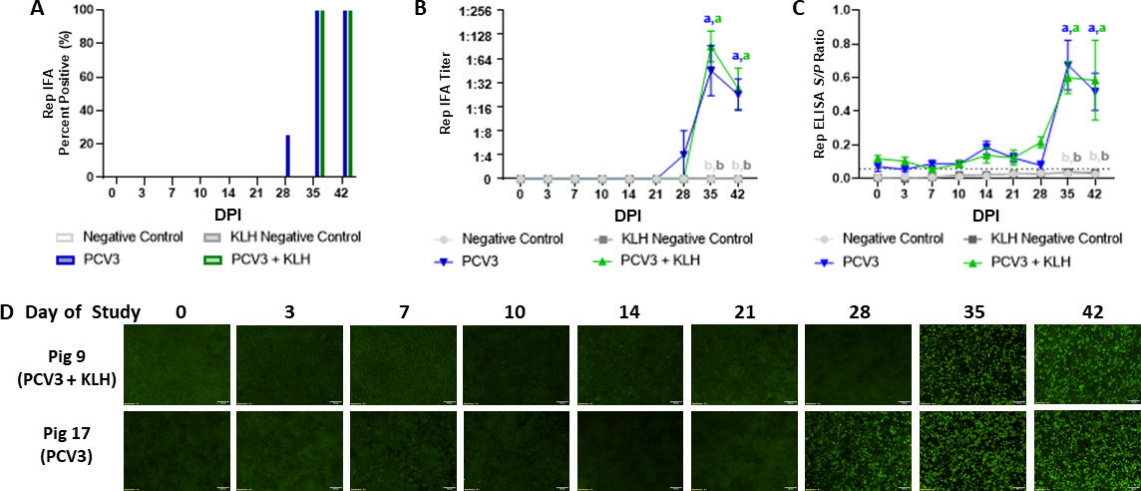
Dynamic of Rep IgG antibodies by IFA and ELISA in CD/CD pigs experimentally in inoculated with PCV3 tissue homogenate with and without immunostimulation. PCV3 and PCV3+KLH groups were inoculated intranasally with 2 mL of PCV3 tissue homogenate (1.04 × 10^11^ genome copies mL^−1^) and intramuscularly with 2 mL of PCV3 tissue homogenate (3.38 × 10^12^ genome copies mL^−1^) then reinoculated 7 days after the first inoculation. Immunostimulation was performed in the PCV3+KLH and KLH negative control groups through subcutaneous administration of 1 mL of 1 mg mL^−1^ of KLH in ICFA. Rep IgG antibodies were evaluated by the percentage of pigs per group (A), average IFA titer by group (B), and average ELISA *S*/*P* ratios by group (C). Seroconversion was detected at 28 dpi with significantly higher antibody levels in the PCV3 and PCV3+KLH groups by IFA and ELISA occurring at 35–42 dpi. (D) The Rep IgG IFA staining from one representative pig in the PCV3 and PCV3+KLH groups at a serum dilution of 1:4 and 4× magnification, where moderate amounts of immunofluorescent staining are present at 28 dpi for pig 17 in the PCV3 group and greater amounts of bright and diffuse immunofluorescent staining from both pigs are present 35–42 dpi, correlating to the increase in IFA titers. Bars represent the SE of the mean, and significance was established at *P* < 0.05.

For the Rep IgG antibody response, a single pig in the PCV3 group had detectable antibodies at 28 dpi by IFA (Fig. 6A and B). All pigs in both PCV3 and PCV3+KLH groups had detectable antibodies by 35 dpi reaching antibody titers detectable by IFA as high as 1:256, which is comparatively 16-fold lower than the maximum Cap IgG IFA titer observed. Furthermore, maximum Rep IgG IFA titers were approximately 30-fold lower than Cap IgG IFA titers at 35 dpi. The IFA titers and ELISA *S*/*P* ratios for the PCV3 and PCV3+KLH group were significantly higher (*P* < 0.05) than the negative control and KLH negative control groups 35–42 dpi (Fig. 6B and C). No significant differences in Rep IgG antibody titers were observed between PCV3 and PCV3+KLH groups by IFA and ELISA at 35 and 42 dpi (Fig. 6B and C). All the pigs in the mock-inoculated negative control and adjuvant control groups were negative for the presence of Rep IgG by IFA and ELISA throughout the study (Fig. 6B and C). The Rep IgG IFA staining of one representative pig from the PCV3 and PCV3+KLH groups is shown in Fig. 6D. Moderate amounts of intracytoplasmic green-fluorescent staining were present at 28 dpi for pig 17 in the PCV3 group. Greater amounts of bright and diffuse immunofluorescent staining from both pigs were present 35–42 dpi, correlating to the increase in IFA titers and ELISA *S*/*P* ratios (Fig. 5D).

Detection and quantitation of PCV3 DNA were assessed throughout the study (data not shown). Briefly, prolonged viremia was observed in the PCV3 and PCV3+KLH groups from 3 dpi to the end of study with no significant changes in viral load regardless of immunostimulation. Peak viremia occurred at 3 dpi with similar average genomic copy numbers per mL^−1^ between the PCV3 (2.20 × 10^8^ copies mL^−1^) and PCV3+KLH groups (1.42 × 10^8^ copies mL^−1^) (28).

### Evaluation of cross-reactivity between PCV3 and PCV2 seropositive sera by IFA and ELISA

**FIG 7:**
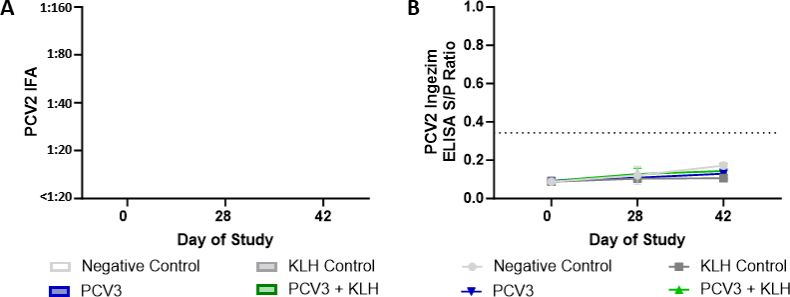
Evaluation of PCV3 antibody cross-reactivity to PCV2 serological methods from pigs experimentally inoculated with PCV3 tissue homogenate. PCV3 and PCV3+KLH groups were inoculated intranasally with 2 mL of PCV3 tissue homogenate (1.04 × 10^11^ genome copies mL^−1^) and intramuscularly with 2 mL of PCV3 tissue homogenate (3.38 × 10^12^ genome copies mL^−1^) then reinoculated 7 days after the first inoculation. Immunostimulation was performed in the PCV3+KLH and KLH negative control groups through subcutaneous administration of 1 mL of 1 mg mL^−1^ of KLH in ICFA. Serum samples were evaluated by PCV2 IFA (A) and Ingezim ELISA (B) conducted at the ISU-VDL. All pigs had a PCV2 IFA titer of <1:20 and PCV2 ELISA *S*/*P* ratios below the positive cutoff, suggesting a negative PCV2 antibody status. Bars represent the SE of the mean.

Sera obtained from PCV3 experimentally infected pigs were evaluated for potential cross-reactivity with PCV2 antigens by different PCV2 serological methods. From study 2, serum samples from all animals at 0, 28, and 42 dpi were evaluated for the presence of viremia and PCV2 antibodies by qPCR, IFA, and ELISA. All animals displayed no PCV2 viremia throughout the study duration (28). Additionally, PCV2 IFA titers were <1:20 for all animals at each time point, consistent with a negative antibody status (Fig. 7A). Furthermore, PCV2 ELISA *S*/*P* ratios fell below the positive cutoff for all animals at each timepoint (Fig. 7B).

**FIG 8:**
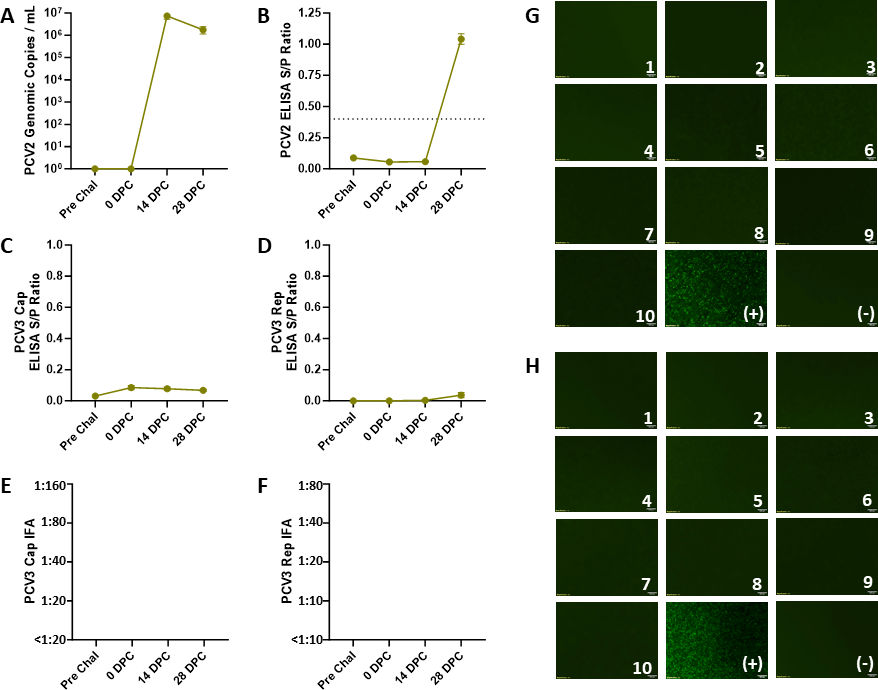
Evaluation of PCV2 antibody cross-reactivity to PCV3 serologic methods from pigs experimentally challenged with PCV2. All pigs were challenged with PCV2 tissue homogenate (2 mL intranasal and 1 mL intramuscular) on 0 dpc. Serum samples were analyzed by PCV2 qPCR (A) and Ingezim ELISA (B) conducted at the ISU-VDL to confirm successful challenge and seroconversion of PCV2 antibodies. Additionally, all pigs had no detectable PCV3 viremia throughout the study duration (data not shown). Serum samples were further evaluated by PCV3 Cap IgG ELISA (C), Rep IgG ELISA (D), Cap IgG IFA (E), and Rep IgG IFA (F). ELISA *S*/*P* ratios remained relatively low, and Cap and Rep IFA titers were <1:20 and <1:10, respectively, suggesting a negative PCV3 antibody status. None of the experimentally treated animals showed staining by PCV3 Cap IgG IFA (G) or Rep IgG IFA (H), in contrast to the positive controls. . Bars represent the SE of the mean.

The presence of PCV2 antibodies following experimental PCV2 infection was evaluated for potential cross-reactivity on PCV3 serological methods developed in this study. For study 3, 10 CD/CD pigs were challenged with PCV2, and serum was collected pre-challenge (49 days before challenge), 0 , 14, and 28 dpc. No PCV2 viremia or PCV2 antibodies were observed in samples collected during pre-challenge (Fig. 8A and B). After challenge, PCV2 viremia was detectable 14 and 28 dpc by qPCR (Fig. 8A), and seroconversion was detected at 28 dpc by ELISA in all animals (Fig. 8B). PCV3 viremia was not detected in all animals throughout the study duration (data not shown). PCV3 Cap and Rep IgG ELISA *S*/*P* ratios were <0.1 at 28 dpc (Fig. 8C and D), supporting the lack of cross-reactivity of PCV2 positive sera on PCV3 ELISA. In addition, PCV3 Cap and Rep IgG IFA titers were <1:20 and <1:10, respectively (Fig. 8E and F), further demonstrating a lack of cross-reactivity of PCV2 antibodies to PCV3 serologic methods. All PCV2 experimentally infected pigs lacked the nuclear green fluorescent staining by the Cap IgG IFA (Fig. 8G) and cytoplasmic green fluorescent staining by the Rep IgG IFA (Fig. 8H) to suggest a positive IFA titer.

## DISCUSSION

Since PCV3 was identified in 2016, the virus has most commonly been associated with multisystemic inflammation, reproductive failure, and subclinical infection (7, 8). Despite the advancements in PCV3 detection and the availability of serological tools for PCV3 serological diagnosis, a significant gap remains in understanding the dynamics of antibodies in grower-finisher pigs, the duration of maternal antibodies, and the potential cross-reactivity with PCV2 antibodies. Field studies have hypothesized the transfer of maternally derived antibodies due to increased detection of viremia in pigs older than 3–4 weeks of age (26, 27). More recently, an experimental infection study in pregnant gilts during mid and late gestion reproduced transplacental infection resulting in piglets with viremia at birth, decreased weaning weight, and lymphohistiocytic arteritis and periarteritis in piglets. Howevere, antibody dynamics were not assessed due to the lack of validated antibody tests (37). Retrospective field studies have reported variable PCV3 seroprevalence ranging from 20% to 80% in varying production phases. However, these studies do not describe the antibody dynamics of natural infection or the transfer of maternally derived antibodies (18, 21). Experimental infection studies have reported conflicting results in the humoral dynamics (22, 28). Furthermore, information regarding the presence and dynamics of PCV3 Rep antibodies remains limited. While cross-reactivity between PCV3 and PCV2 has been explored by *in vitro* analyses with field samples, controlled experiments utilizing experimentally infected animals to elucidate this diagnostic inquiry are lacking. Thus, this study aims to address these three primary knowledge gaps concerning the humoral response to PCV3 and the serological diagnosis of PCV3.

**FIG 9:**
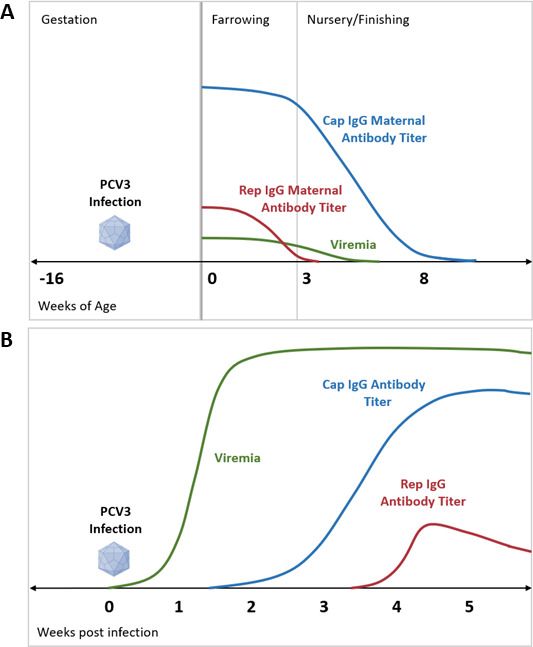
Summary of PCV3 viremia, Cap IgG, and Rep IgG dynamics following maternal antibody transfer in naturally PCV3 infected sows (A) and experimental PCV3 infection (B).

The transfer of PCV2 maternal antibodies has been well characterized where both PCV2 Cap and Rep maternally derived antibodies can be detected in piglets (29). The half-life of PCV2 Cap maternally derived antibodies is approximately 19 days (38). However, the duration of PCV2 Cap antibodies in piglets can vary depending on the maternal antibody levels. Thus, based on the immunological status of the sow, the dynamics of low, mid, and high maternal antibody transfer can last approximately 4–6, 6–10, and 8.5–13 weeks of age, respectively (38). The presence of PCV3 maternal antibodies has yet to be fully evaluated. The presence of PCV3 maternally derived antibodies has been hypothesized due to a lack of detectable viremia during the lactation phase and increased viral detection in pigs older than 3–4 weeks (26, 27). In the present study, 30 sows from a commercial farm with a history of PCV3 were evaluated for the presence of Cap antibody 2 weeks before farrowing. Three litters from PCV3 antibody positive sows were selected for longitudinal evaluation of the Cap and Rep IgG humoral response 1–9 weeks post-farrowing. All piglets from all litters displayed the highest levels of PCV3 Cap IgG antibodies 1-week post-farrowing. These antibodies waned to nondetectable levels by approximately 6–8 weeks post-farrowing for litters 1 and 3, while antibodies were detectable in approximately 60% of the litter 2 piglets by IFA at 9 weeks post-farrowing (Fig. 9A). Multiple factors influence maternal antibody transfer including piglet milk intake, milk availability, the immune status of the sows, sow parity, and husbandry practices aimed at enhancing colostrum intake (39, 40). As per these findings, the dynamic of PCV3 Cap maternally derived antibodies was similar to the transfer of a moderate quantity of PCV2 Cap-specific maternal antibodies (38). Further studies are necessary to evaluate how differences in sow antibody levels may affect the duration of the Cap antibody dynamic in piglets.

In accordance with previous studies assessing the PCV2 Rep IgG maternal antibodies, maternally derived PCV2 Rep IgG antibodies have been detected until 11 weeks of age in piglets (29). However, the approximate duration of PCV2 Rep antibodies has not been characterized in terms of sow antibody levels. In this study, PCV3 Rep IgG antibody detection was more variable between litters. Maternally derived PCV3 Rep IgG antibodies were not detected in litter 1, while antibodies waned by approximately 5-weeks post-farrowing in litters 2 and 3. Although the duration of PCV3 Rep IgG antibodies can also be associated with several factors similar to those reported affecting PCV2 Cap antibody duration (39, 40), our results demonstrate that lower amounts of maternally derived PCV3 Rep antibodies were transferred to the piglets compared to Cap antibodies. Additionally, our results may suggest that PCV3 Rep antibodies have a shorter half-life compared to PCV3 Cap antibodies and the PCV3 Rep antibody dynamic may be shorter than PCV2 Rep antibodies (29).

During PCV2 infection, clinical PCVAD is generally not observed before 4 weeks of age due to the presence of maternally derived antibodies (41–44). Thus, PCV3 maternally antibodies may have a similar role in protecting against infection in the lactation and nursery phase. In previous field studies, pigs 3–4 weeks of age displayed no PCV3 viremia (26). Furthermore, the proportion of PCV3 viremia detection increased in pigs from 6 to 24 weeks of age (27). The present study did not examine whether piglets developed lesions compatible with PCV3 infection. Thus, the study cannot confirm that the presence of maternally derived antibodies provided complete protection against infection. However, piglets did not develop apparent clinical signs suggestive of PCV3 infection, indicating that maternally derived antibodies may have aided in the clearance of viremia during 1–4-weeks post–farrowing, resulting in subclinical infection. Interestingly, viremia was detected in litter 3 at 9-weeks post-farrowing, in concordance with the absence of maternally derived antibodies, potentially suggesting the detection of an early infection. Future studies should examine when the development of viremia occurs in relationship to low, mid, and high levels of PCV3 maternal antibody transfer.

The antibody response following PCV2 infection has also been well characterized where seroconversion occurs 7–28 dpi and neutralizing antibodies are detectable beginning at 10 dpi (30). Furthermore, Cap antibodies are produced earlier and reach higher titer compared to Rep antibodies (31). In the present study, the Cap and Rep IgG responses were evaluated following experimental inoculation with PCV3 tissue homogenate with and without immunostimulant. Regardless of immunostimulation, the PCV3 Cap IgG response was detectable in both inoculated PCV3 groups at 14 dpi reaching highest levels detected either by IFA or by ELISA by 28–42 dpi (Fig. 9B). ELISA *S*/*P* ratios from 28 to 42 dpi showed that immune stimulation with KLH induced significantly higher levels of antibodies (*P* < 0.05) compared to the PCV3 group. These significant differences in antibody titers between inoculated groups were not observed by IFA. This is perhaps due to intrinsic factors of the IFA technique such as the two-fold serial dilution of sera, potentially resulting in a less precise determination of titer at higher dilutions. Several previous PCV2 studies have shown immunostimulation by a variety of methods such as adjuvants, vaccination, or coinfection agents triggering the development of PCVAD (45). Thus, based on our results, PCV3 immunostimulation may enhance the Cap IgG humoral response, suggesting co-factors such as coinfections may also exacerbate the PCV3 humoral response. Additional studies evaluating the humoral response to PCV3 and other coinfections are necessary to not only to evaluate the PCV3 antibody dynamics but also to characterize the potential effect of PCV3 infection on the immune response against other pathogens.

In the present study, PCV3 Rep IgG antibodies were detectable 28 dpi with significantly higher IFA titers and ELISA *S*/*P* ratios occurring at 35–42 dpi (Fig. 9B). Interestingly, the highest Rep IgG IFA titers were approximately 30-fold lower than Cap IgG IFA titers at 35 dpi. These results show PCV3 Cap antibodies are produced earlier and at much greater amounts compared to PCV3 Rep antibodies, which coincided with the levels and the dynamic previously reported for PCV2 humoral response (31). The PCV2 Rep protein is a weak immunogen. Vaccination with Rep DNA vaccines followed by a booster with recombinant protein did not result in protection against clinical disease. In comparison, a DNA Cap vaccine following the same vaccination and challenge model abrogated the development of clinical disease, confirming the Cap protein of PCV2 is the major protective immunogen (46). Similar to PCV3, the PCV2 Cap is the sole component of the virion. Therefore, the PCV2 Rep protein is expressed following initial viral replication mediated by host cell DNA replication machinery (47). Much is still unknown regarding the PCV3 life cycle. However, given the similarities of PCV2 and PCV3, the intracytoplasmic nature of the Rep protein and reliance on the host cell for expression may explain the delayed development of Rep antibodies at lower levels, which may potentially provide limited protection. The biological activity of Rep maternally derived antibodies and Rep antibodies developed following infection should be further evaluated.

Interestingly, prolonged PCV3 viremia was observed in the PCV3 and PCV3+KLH groups from 3 dpi to the end of study with no significant changes in viral load regardless of immunostimulation (28). The development and maintenance of prolonged viremia while pigs produce an antibody response is a similar feature of infection for PCV2 and PCV3. (48–50). Following PCV2 infection, virus-specific neutralizing antibodies are detectable at approximately 21 dpi (41, 45, 51, 52). Virus-neutralizing antibodies appear to limit viral replication *in vitro* (30, 53) and the development of PCVAD *in vivo* (54). However, protective immunity following vaccination can be achieved with low levels of humoral immunity (55), suggesting both the humoral and cell-mediated immune responses contribute to protection (56). Pigs naturally infected with PCV2 develop a robust Interferon‐gamma (IFN‐γ) response (57). Furthermore, PCV2-specific IFN-γ production has been correlated to lower levels of viremia (58, 59), demonstrating the cell-mediated response is vital for clearance of PCV2 infection (56, 60–63). Future studies should aim to determine the duration of the PCV3 antibody response, development of neutralizing antibodies, and potential correlation of total and virus-specific neutralizing antibody titers to the reduction of viremia. Additionally, future research should elucidate the development of PCV3-specific INF-γ production.

Because of limited sample volume, the current study was limited to focusing on characterizing the Cap and Rep IgG dynamics. However, characterizing the IgM response warrants further investigation. Previous experimental PCV3 inoculation studies have conflicting results on the presence of Cap IgM (22, 28). For PCV2 infection, the presence of the Cap IgM isotype seems to be an indicator of the development of clinical disease. In pigs affected with postweaning multisystemic wasting syndrome (PMWS), IgM remained at low or nondetectable titers and IgG titers were significantly lower compared to healthy group mates (30). Furthermore, the IgM isotype is correlated to low levels of neutralizing activity and has no correlation to viremia reduction within the first 2 weeks of PCV2 infection (64). Therefore, we hypothesize that the presence of IgM may be correlated to PCV3 clinical disease and the persistence of IgM in the absence of class switching to the IgG isotype could result in the development of PCV3 clinical disease.

The genetic homology of PCV2 and PCV3 is approximately 46.8% at the whole genome level, 26%–36% for the ORF2, and 48% for the ORF1 (8, 12, 16). Despite the low genomic similarity, potential antibody cross-reactivity between PCV2 and PCV3 has been proposed, and information generated based on *in vitro* studies is nonconclusive. Convalescent rabbit serum generated by the immunization of recombinant PCV2-, PCV3-, and PCV4-truncated Cap proteins showed cross-reactivity among all the three PCVs using viral-based IFAs using virus rescued from infectious clones. Cross-reactivity was hypothesized due to the presence of a conserved epitope within the PCV2, PCV3, and PCV4 Cap genes (65). However, several studies have failed to recapitulate the *in vitro* cross-reactivity of PCV2 and PCV3 antibodies using swine convalescent serum. PCV2 antibody positive serum was not cross reactive to a PCV3 IFA by expression of the PCV3 Cap in a baculovirus system (24). Furthermore, PCV3 convalescent serum has shown no cross-reactivity to PCV2 virus-like particles (21). The current study further characterizes the cross-reactivity of PCV2 and PCV3 antibodies in convalescent serum from experimentally infected animals. No cross-reactivity of PCV3 antibodies was observed by PCV2 IFA and ELISA. Furthermore, no cross-reactivity of PCV2 antibodies was displayed by PCV3 Cap and Rep IFA and ELISA. These findings may indicate that natural infection may not confer protection against the alternate PCV through the humoral response. Future investigations ought to validate the absence of cross-protection offered by either natural infection or vaccination against the alternate PCV.

Characterization of the PCV2 protective immunity provided by maternal antibody transfer, and dynamic following natural infection has resulted in the development of highly implemented vaccination schedules to greatly reduce PCVAD and subclinical infection (66). While no vaccines are currently commercially available for PCV3, further research into the neutralizing antibody response and the protection provided following maternal antibody transfer and natural infection may aid in the establishment of husbandry practices and potential future application of prophylactics to control PCV3 clinical disease.

## References

[1] Tischer I, Gelderblom H, Vettermann W, Koch MA. 1982. A very small porcine virus with circular single-stranded DNA. Nature 295:64–66. doi:10.1038/295064a0 7057875

[2] Tischer I, Rasch R, Tochtermann G. 1974. Characterization of papovavirus and picornavirus-like particles in permanent pig kidney cell lines. Zentralbl Bakteriol Orig A 226:153–167. 4151202

[3] Tischer I, Mields W, Wolff D, Vagt M, Griem W. 1986. Studies on epidemiology and pathogenicity of porcine circovirus. Arch Virol 91:271–276. doi:10.1007/BF01314286 3778212

[4] Allan GM, McNeilly F, Kennedy S, Daft B, Clarke EG, Ellis JA, Haines DM, Meehan BM, Adair BM. 1998. Isolation of porcine circovirus-like viruses from pigs with a wasting disease in the USA and Europe. J Vet Diagn Invest 10:3–10. doi:10.1177/104063879801000102 9526853

[5] Ellis J, Hassard L, Clark E, Harding J, Allan G, Willson P, Strokappe J, Martin K, McNeilly F, Meeran B, Todd D, Haines D. 1998. Isolation of circovirus from lesions of pigs with postweaning multisystemic wasting syndrome. Can Vet J 39:44–51. PMC1539838 9442952

[6] Segalés J. 2012. Porcine circovirus type 2 (PCV2) infections: clinical signs, pathology and laboratory diagnosis. Virus Res 164:10–19. doi:10.1016/j.virusres.2011.10.007 22056845

[7] Kroeger M, Temeeyasen G, Piñeyro PE. 2022. Five years of porcine circovirus 3: what have we learned about the clinical disease, immune pathogenesis, and diagnosis. Virus Res 314:198764. doi:10.1016/j.virusres.2022.198764 35367483

[8] Palinski R, Piñeyro P, Shang P, Yuan F, Guo R, Fang Y, Byers E, Hause BM. 2017. A novel porcine circovirus distantly related to known circoviruses is associated with porcine dermatitis and nephropathy syndrome and reproductive failure. J Virol 91:e01879-16. doi:10.1128/JVI.01879-16 27795441 PMC5165205

[9] Zhang H-H, Hu W-Q, Li J-Y, Liu T-N, Zhou J-Y, Opriessnig T, Xiao C-T. 2020. Novel circovirus species identified in farmed pigs designated as porcine circovirus 4, Hunan province, China. Transbound Emerg Dis 67:1057–1061. doi:10.1111/tbed.13446 31823481

[10] Holgado-Martín R, Arnal JL, Sibila M, Franzo G, Martín-Jurado D, Risco D, Segalés J, Gómez L. 2023. First detection of porcine circovirus 4 (PCV-4) in Europe. Virol J 20:230. doi:10.1186/s12985-023-02181-1 37817216 PMC10566016

[11] Finsterbusch T, Steinfeldt T, Caliskan R, Mankertz AJV. 2005. Analysis of the subcellular localization of the proteins Rep, Rep′ and Cap of porcine circovirus type 1. Virology 343:36–46. doi:10.1016/j.virol.2005.08.021 16168452

[12] Phan TG, Giannitti F, Rossow S, Marthaler D, Knutson TP, Li L, Deng X, Resende T, Vannucci F, Delwart E. 2016. Detection of a novel circovirus PCV3 in pigs with cardiac and multi-systemic inflammation. Virol J 13:184. doi:10.1186/s12985-016-0642-z 27835942 PMC5105309

[13] Mankertz A, Domingo M, Folch JM, LeCann P, Jestin A, Segalés J, Chmielewicz B, Plana-Durán J, Soike D. 2000. Characterisation of PCV-2 isolates from Spain, Germany and France. Virus Res 66:65–77. doi:10.1016/s0168-1702(99)00122-7 10653918

[14] Niu G, Chen S, Li X, Zhang L, Ren L. 2022. Advances in crosstalk between porcine circoviruses and host. Viruses 14:1419. doi:10.3390/v14071419 35891399 PMC9315664

[15] Mankertz A, Mankertz J, Wolf K, Buhk HJ. 1998. Identification of a protein essential for replication of porcine circovirus. J Gen Virol 79:381–384. doi:10.1099/0022-1317-79-2-381 9472624

[16] Guo Z, Li X, Deng R, Zhang G. 2019. Detection and genetic characteristics of porcine circovirus 3 based on oral fluids from asymptomatic pigs in central China. BMC Vet Res 15:200. doi:10.1186/s12917-019-1952-3 31196107 PMC6567530

[17] Maity HK, Samanta K, Deb R, Gupta VK. 2023. Revisiting porcine circovirus infection: recent insights and its significance in the piggery sector. Vaccines (Basel) 11:1308. doi:10.3390/vaccines11081308 37631876 PMC10457769

[18] Zhang S, Wang D, Jiang Y, Li Z, Zou Y, Li M, Yu H, Huang K, Yang Y, Wang N. 2019. Development and application of a baculovirus-expressed capsid protein-based indirect ELISA for detection of porcine circovirus 3 IgG antibodies. BMC Vet Res 15:79. doi:10.1186/s12917-019-1810-3 30841883 PMC6404275

[19] Wang Y, Wang G, Duan W-T, Sun M-X, Wang M-H, Wang S-H, Cai X-H, Tu Y-B. 2020. Self-assembly into virus–like particles of the recombinant capsid protein of porcine circovirus type 3 and its application on antibodies detection. AMB Express 10:3. doi:10.1186/s13568-019-0940-0 31912330 PMC6946787

[20] Liu BY, Gao B, Liu MZ, Zhang TT, Liu BS, Chen ZL. 2020. High repetitive arginine in the anterior of PCV3 capsid protein is a severe obstacle for its expression in E. coli. AMB Express 10:214. doi:10.1186/s13568-020-01163-8 33306160 PMC7732928

[21] Deng J, Li X, Zheng D, Wang Y, Chen L, Song H, Wang T, Huang Y, Pang W, Tian K. 2018. Establishment and application of an indirect ELISA for porcine circovirus 3. Arch Virol 163:479–482. doi:10.1007/s00705-017-3607-7 29079953

[22] Mora-Díaz J, Piñeyro P, Shen H, Schwartz K, Vannucci F, Li G, Arruda B, Giménez-Lirola L. 2020. Isolation of PCV3 from perinatal and reproductive cases of PCV3-associated disease and in vivo characterization of PCV3 replication in CD/CD growing pigs. Viruses 12:219. doi:10.3390/v12020219 32079070 PMC7077311

[23] Geng S, Luo H, Liu Y, Chen C, Xu W, Chen Y, Li X, Fang W. 2019. Prevalence of porcine circovirus type 3 in pigs in the southeastern Chinese province of Zhejiang. BMC Vet Res 15:244. doi:10.1186/s12917-019-1977-7 31307451 PMC6631677

[24] Yao L, Li C, Wang J, Cheng Y, Ghonaim AH, Sun Q, Yu X, Niu W, Fan S, He Q. 2021. Development of an indirect immunofluorescence assay for PCV3 antibody detection based on capsid protein. Anim Dis 1:1–8. doi:10.1186/s44149-021-00015-7

[25] Jiang M, Guo J, Zhang G, Jin Q, Liu Y, Jia R, Wang A. 2020. Fine mapping of linear B cell epitopes on capsid protein of porcine circovirus 3. Appl Microbiol Biotechnol 104:6223–6234. doi:10.1007/s00253-020-10664-2 32445000

[26] Stadejek T, Woźniak A, Miłek D, Biernacka K. 2017. First detection of porcine circovirus type 3 on commercial pig farms in Poland. Transbound Emerg Dis 64:1350–1353. doi:10.1111/tbed.12672 28649803

[27] Klaumann F, Correa-Fiz F, Sibila M, Núñez JI, Segalés J. 2019. Infection dynamics of porcine circovirus type 3 in longitudinally sampled pigs from four Spanish farms. Vet Rec 184:619–619. doi:10.1136/vr.105219 31040218

[28] Temeeyasen G, Lierman S, Arruda BL, Main R, Vannucci F, Gimenez-Lirola LG, Piñeyro PE. 2021. Pathogenicity and immune response against porcine circovirus type 3 infection in caesarean-derived, colostrum-deprived pigs. J Gen Virol 102. doi:10.1099/jgv.0.001502 33206034

[29] Pérez-Martín E, Grau-Roma L, Argilaguet JM, Nofrarías M, Escribano JM, Gómez-Sebastián S, Segalés J, Rodríguez F. 2008. Development of two Trichoplusia ni larvae-derived ELISAs for the detection of antibodies against replicase and capsid proteins of porcine circovirus type 2 in domestic pigs. J Virol Methods 154:167–174. doi:10.1016/j.jviromet.2008.07.034 18773923

[30] Meerts P, Misinzo G, Lefebvre D, Nielsen J, Bøtner A, Kristensen CS, Nauwynck HJ. 2006. Correlation between the presence of neutralizing antibodies against porcine circovirus 2 (PCV2) and protection against replication of the virus and development of PCV2-associated disease. BMC Vet Res 2:6. doi:10.1186/1746-6148-2-6 16445856 PMC1386657

[31] Pogranichnyy RM, Yoon KJ, Harms PA, Swenson SL, Zimmerman JJ, Sorden SD. 2000. Characterization of immune response of young pigs to porcine circovirus type 2 infection. Viral Immunol 13:143–153. doi:10.1089/vim.2000.13.143 10892995

[32] Houston E, Giménez-Lirola LG, Magtoto R, Mora-Díaz JC, Baum D, Piñeyro PE. 2019. Seroprevalence of Senecavirus A in sows and grower-finisher pigs in major swine producing-states in the United States. Prev Vet Med 165:1–7. doi:10.1016/j.prevetmed.2019.01.012 30851922

[33] Thrusfield MJ. 1997. Veterinary epidemiology, p 41–42. In Blackwell science, 2nd ed. Oxford.

[34] Arruda B, Piñeyro P, Derscheid R, Hause B, Byers E, Dion K, Long D, Sievers C, Tangen J, Williams T, Schwartz K. 2019. PCV3-associated disease in the United States swine herd. Emerg Microbes Infect 8:684–698. doi:10.1080/22221751.2019.1613176 31096848 PMC6534263

[35] Okda F, Lawson S, Liu X, Singrey A, Clement T, Hain K, Nelson J, Christopher-Hennings J, Nelson EA. 2016. Development of monoclonal antibodies and serological assays including indirect ELISA and fluorescent microsphere immunoassays for diagnosis of porcine deltacoronavirus. BMC Vet Res 12:95. doi:10.1186/s12917-016-0716-6 27277214 PMC4898321

[36] Classen DC, Morningstar JM, Shanley JD. 1987. Detection of antibody to murine cytomegalovirus by enzyme-linked immunosorbent and indirect immunofluorescence assays. J Clin Microbiol 25:600–604. doi:10.1128/jcm.25.4.600-604.1987 3033015 PMC266042

[37] Cobos À, Ruiz A, Pérez M, Llorens A, Huerta E, Correa-Fiz F, Lohse R, Balasch M, Segalés J, Sibila M. 2023. Experimental inoculation of porcine circovirus 3 (PCV-3) in pregnant gilts causes PCV-3-associated lesions in newborn piglets that persist until weaning. Transboundary Emerging Dis 2023:1–14. doi:10.1155/2023/5270254 PMC12016758 40303807

[38] Opriessnig TYS, Thacker EL, Halbur PG. 2004. Derivation of porcine circovirus type 2-negative pigs from positive breeding herds. J Swine Health Prod 12:186–191.

[39] Martínez-Boixaderas N, Garza-Moreno L, Sibila M, Segalés J. 2022. Impact of maternally derived immunity on immune responses elicited by piglet early vaccination against the most common pathogens involved in porcine respiratory disease complex. Porcine Health Manag 8:11. doi:10.1186/s40813-022-00252-3 35296365 PMC8928644

[40] Klobasa F, Werhahn E, Butler JE. 1981. Regulation of humoral immunity in the piglet by immunoglobulins of maternal origin. Res Vet Sci 31:195–206. doi:10.1016/S0034-5288(18)32494-9 7323466

[41] McKeown NE, Opriessnig T, Thomas P, Guenette DK, Elvinger F, Fenaux M, Halbur PG, Meng XJ. 2005. Effects of porcine circovirus type 2 (PCV2) maternal antibodies on experimental infection of piglets with PCV2. Clin Diagn Lab Immunol 12:1347–1351. doi:10.1128/CDLI.12.11.1347-1351.2005 16275955 PMC1287757

[42] Ostanello F, Caprioli A, Di Francesco A, Battilani M, Sala G, Sarli G, Mandrioli L, McNeilly F, Allan GM, Prosperi S. 2005. Experimental infection of 3-week-old conventional colostrum-fed pigs with porcine circovirus type 2 and porcine parvovirus. Vet Microbiol 108:179–186. doi:10.1016/j.vetmic.2005.04.010 15916871

[43] McIntosh KA, Harding JCS, Ellis JA, Appleyard GD. 2006. Detection of porcine circovirus type 2 viremia and seroconversion in naturally infected pigs in a farrow-to-finish barn. Can J Vet Res 70:58–61. 16548333 PMC1325095

[44] Gillespie J, Opriessnig T, Meng XJ, Pelzer K, Buechner-Maxwell V. 2009. Porcine circovirus type 2 and porcine circovirus-associated disease. J Vet Intern Med 23:1151–1163. doi:10.1111/j.1939-1676.2009.0389.x 19780932 PMC7166794

[45] Meng XJ. 2013. Porcine circovirus type 2 (PCV2): pathogenesis and interaction with the immune system. Annu Rev Anim Biosci 1:43–64. doi:10.1146/annurev-animal-031412-103720 25387012

[46] Blanchard P, Mahé D, Cariolet R, Keranflec’h A, Baudouard MA, Cordioli P, Albina E, Jestin A. 2003. Protection of swine against post-weaning multisystemic wasting syndrome (PMWS) by porcine circovirus type 2 (PCV2) proteins. Vaccine 21:4565–4575. doi:10.1016/s0264-410x(03)00503-6 14575769

[47] Franzo G, Segalés J. 2021. Circoviruses (*Circoviridae*), p 182–192. In Bamford DH, Zuckerman M (ed), Encyclopedia of virology, Fourth Edition. Academic Press, Oxford.

[48] Larochelle R, Magar R, D’Allaire S. 2003. Comparative serologic and virologic study of commercial swine herds with and without postweaning multisystemic wasting syndrome. Can J Vet Res 67:114–120. 12760476 PMC227038

[49] Rodríguez-Arrioja GM, Segalés J, Calsamiglia M, Resendes AR, Balasch M, Plana-Duran J, Casal J, Domingo M. 2002. Dynamics of porcine circovirus type 2 infection in a herd of pigs with postweaning multisystemic wasting syndrome. Am J Vet Res 63:354–357. doi:10.2460/ajvr.2002.63.354 11911570

[50] Sibila M, Calsamiglia M, Segalés J, Blanchard P, Badiella L, Le Dimna M, Jestin A, Domingo M. 2004. Use of a polymerase chain reaction assay and an ELISA to monitor porcine circovirus type 2 infection in pigs from farms with and without postweaning multisystemic wasting syndrome. Am J Vet Res 65:88–92. doi:10.2460/ajvr.2004.65.88 14719708

[51] Beach NM, Ramamoorthy S, Opriessnig T, Wu SQ, Meng XJ. 2010. Novel chimeric porcine circovirus (PCV) with the capsid gene of the emerging PCV2b subtype cloned in the genomic backbone of the non-pathogenic PCV1 is attenuated in vivo and induces protective and cross-protective immunity against PCV2b and PCV2a subtypes in pigs. Vaccine 29:221–232. doi:10.1016/j.vaccine.2010.10.050 21044670

[52] Beach NM, Smith SM, Ramamoorthy S, Meng X-J. 2011. Chimeric porcine circoviruses (PCV) containing amino acid epitope tags in the C terminus of the capsid gene are infectious and elicit both anti-epitope tag antibodies and anti-PCV type 2 neutralizing antibodies in pigs. J Virol 85:4591–4595. doi:10.1128/JVI.02294-10 21307200 PMC3126229

[53] Meerts P, Van Gucht S, Cox E, Vandebosch A, Nauwynck HJ. 2005. Correlation between type of adaptive immune response against porcine circovirus type 2 and level of virus replication. Viral Immunol 18:333–341. doi:10.1089/vim.2005.18.333 16035945

[54] Song Y, Jin M, Zhang S, Xu X, Xiao S, Cao S, Chen H. 2007. Generation and immunogenicity of a recombinant pseudorabies virus expressing cap protein of porcine circovirus type 2. Vet Microbiol 119:97–104. doi:10.1016/j.vetmic.2006.08.026 17005335

[55] Fenaux M, Opriessnig T, Halbur PG, Elvinger F, Meng XJ. 2004. A chimeric porcine circovirus (PCV) with the immunogenic capsid gene of the pathogenic PCV type 2 (PCV2) cloned into the genomic backbone of the nonpathogenic PCV1 induces protective immunity against PCV2 infection in pigs. J Virol 78:6297–6303. doi:10.1128/JVI.78.12.6297-6303.2004 15163723 PMC416547

[56] Fort M, Sibila M, Pérez-Martín E, Nofrarías M, Mateu E, Segalés J. 2009. One dose of a porcine circovirus 2 (PCV2) sub-unit vaccine administered to 3-week-old conventional piglets elicits cell-mediated immunity and significantly reduces PCV2 viremia in an experimental model. Vaccine 27:4031–4037. doi:10.1016/j.vaccine.2009.04.028 19379787

[57] Ferrari L, Borghetti P, De Angelis E, Martelli P. 2014. Memory T cell proliferative responses and IFN-γ productivity sustain long-lasting efficacy of a cap-based PCV2 vaccine upon PCV2 natural infection and associated disease. Vet Res 45:44. doi:10.1186/1297-9716-45-44 24735253 PMC3999888

[58] Park C, Seo HW, Han K, Chae CJC, Immunology V. 2014. Comparison of four commercial one-dose porcine circovirus type 2 (PCV2) vaccines administered to pigs challenged with PCV2 and porcine reproductive and respiratory syndrome virus at 17 weeks postvaccination to control porcine respiratory disease complex under Korean field conditions. Clin Vaccine Immunol 21:399–406. doi:10.1128/CVI.00768-13 24403524 PMC3957680

[59] Seo HW, Lee J, Han K, Park C, Chae C. 2014. Comparative analyses of humoral and cell-mediated immune responses upon vaccination with different commercially available single-dose porcine circovirus type 2 vaccines. Res Vet Sci 97:38–42. doi:10.1016/j.rvsc.2014.04.007 24794246

[60] Fort M, Fernandes LT, Nofrarias M, Díaz I, Sibila M, Pujols J, Mateu E, Segalés J. 2009. Development of cell-mediated immunity to porcine circovirus type 2 (PCV2) in caesarean-derived, colostrum-deprived piglets. Vet Immunol Immunopathol 129:101–107. doi:10.1016/j.vetimm.2008.12.024 19167096 PMC7127047

[61] Kim D, Kim CH, Han K, Seo HW, Oh Y, Park C, Kang I, Chae C. 2011. Comparative efficacy of commercial Mycoplasma hyopneumoniae and porcine circovirus 2 (PCV2) vaccines in pigs experimentally infected with M. hyopneumoniae and PCV2. Vaccine 29:3206–3212. doi:10.1016/j.vaccine.2011.02.034 21354247

[62] Oh Y, Seo HW, Han K, Park C, Chae C. 2012. Protective effect of the maternally derived porcine circovirus type 2 (PCV2)-specific cellular immune response in piglets by dam vaccination against PCV2 challenge. J Gen Virol 93:1556–1562. doi:10.1099/vir.0.041749-0 22495234

[63] Opriessnig T, Patterson AR, Madson DM, Pal N, Halbur PG. 2009. Comparison of efficacy of commercial one dose and two dose PCV2 vaccines using a mixed PRRSV–PCV2–SIV clinical infection model 2–3-months post vaccination. Vaccine 27:1002–1007. doi:10.1016/j.vaccine.2008.11.105 19100807

[64] Opriessnig T, Prickett JR, Madson DM, Shen H-G, Juhan NM, Pogranichniy RR, Meng X-J, Halbur PG. 2010. Porcine circovirus type 2 (PCV2)-infection and re-inoculation with homologous or heterologous strains: virological, serological, pathological and clinical effects in growing pigs. Vet Res 41:31. doi:10.1051/vetres/2010003 20167193 PMC2826091

[65] Ji W, Zhang X, Niu G, Chen S, Li X, Yang L, Zhang L, Ren L. 2022. Expression and immunogenicity analysis of the capsid proteins of porcine circovirus types 2 to 4. Int J Biol Macromol 218:828–838. doi:10.1016/j.ijbiomac.2022.07.204 35907450

[66] Franzo G, Segalés J. 2020. Porcine circovirus 2 genotypes, immunity and vaccines: multiple genotypes but one single serotype. Pathogens 9:1049. doi:10.3390/pathogens9121049 33327478 PMC7764931

